# Association between systemic immune-inflammation index and ICU-acquired infection in critically Ill patients without infection: a competing risks analysis from a retrospective cohort

**DOI:** 10.1186/s12879-026-13568-0

**Published:** 2026-05-18

**Authors:** Yulian Ren, Lizhong Jing, Dongqing Hu, Lu Li, Tian Liu, Xianqing Meng, Xiao Ji

**Affiliations:** 1 Department of Infection Management, Shandong University of Traditional Chinese Medicine Affiliated Hospital, No. 16369 Jingshi Road, Jinan City, Shandong Province China; 2 Department of Hepatology, Shandong University of Traditional Chinese Medicine Affiliated Hospital, Jinan, Shandong China; 3 Department of Sports Medicine, Shandong University of Traditional Chinese Medicine Affiliated Hospital, Jinan, Shandong China; 4 Tumor Comprehensive Third Ward (Interventional Minimally Invasive), Shandong Provincial Public Health Clinical Center, Jinan, Shandong China

**Keywords:** Systemic immune-inflammation index, Intensive care unit, Hospital-acquired infection, Biomarker, Risk stratification

## Abstract

**Background:**

Healthcare-associated infections and their complications represent a predominant cause of morbidity and mortality in intensive care units (ICUs) globally. Several studies have reported that the systemic immune-inflammation index (SII) is associated with an increased risk of infection; however, its association with ICU-acquired infection (ICU-AI) remains unclear. We aimed to examine the association between SII and the subsequent risk of ICU-AI.

**Methods:**

We conducted a retrospective cohort study using the MIMIC-IV database, including adult ICU patients without suspected infection at baseline. SII was calculated as platelet × neutrophil / lymphocyte count, based on values from the first 24 h of ICU admission. The primary outcome was ICU-AI, defined as suspected infection occurring after 48 h of ICU admission. Two competing risk regression models were employed to evaluate the association between SII and ICU-AI.

**Results:**

Among the 5,459 patients included, 627 (11.5%) developed ICU-AI. Higher SII (per 1,000-unit) was independently associated with an increased risk of ICU-AI in both models: adjusted cause-specific hazard ratio (CSHR), 1.06 (95% CI, 1.03–1.08; *p* < 0.001) and subdistribution hazard ratio (SHR), 1.05 (95% CI, 1.03–1.07; *p* < 0.001). When analyzed by quartiles, a significant dose-response relationship was observed (p for trend < 0.001). Compared with patients in the lowest quartile (Q1, SII ≤ 594 × 10⁹/L), those in Q3 (SII 1125–2201 × 10⁹/L) and Q4 (SII ≥ 2202 × 10⁹/L) showed progressively higher risks of infection, with adjusted CSHR of 1.44 (95% CI 1.11–1.86) and 1.76 (95% CI 1.37–2.26), and adjusted SHR of 1.51 (95% CI 1.17–1.93) and 1.96 (95% CI 1.54–2.49), respectively. Time-to-event curves showed distinct and consistent cumulative risk trajectories across SII quartiles.

**Conclusion:**

Elevated SII at ICU admission was independently associated with an increased risk of developing suspected secondary infection in infection-naïve critically ill patients.

**Supplementary Information:**

The online version contains supplementary material available at 10.1186/s12879-026-13568-0.

## Introduction

Healthcare-associated infections (HAIs) and their related complications, including sepsis and septic shock, remain among the leading causes of morbidity and mortality in intensive care units (ICUs) worldwide [1]. Global data indicate that approximately 54% of ICU patients present with suspected or confirmed infections, among which ICU-acquired infections (ICU-AI) account for nearly 22%. ICU-AI is associated with a mortality rate exceeding 30%, representing a 32% higher risk of death compared to community-acquired infection [2]. Beyond short-term adverse outcomes, survivors of ICU-AI frequently exhibit persistent immune dysfunction and face an increased risk of mortality that may extend up to 1 year after infection [3]. Despite significant advances in critical care and infection control over recent years, the incidence of HAIs remains unacceptably high, underscoring the need for more effective risk assessment and intervention strategies.

Early identification of patients at high risk for ICU-AI is essential to facilitate individualized infection surveillance and preventive measures. Risk factors for ICU-AI in critically ill patients include advanced age, pre-existing comorbidities, immunosuppression, disease severity, prolonged hospitalization, and exposure to invasive devices [4–6]. Among these, immune dysregulation is increasingly recognized as a pivotal intrinsic mechanism. Critically ill patients often exhibit a biphasic immune response, characterized initially by hyperinflammation (e.g., cytokine storm), followed by immune suppression marked by impaired immune cell function such as T-cell anergy and neutrophil dysfunction [7, 8]. This dysregulated immune profile has been closely linked to the development of secondary infections and poor clinical outcomes [9, 10]. Recent transcriptomic studies further demonstrate that ICU-AI patients display a distinct blood signature early after ICU admission, characterized by activation of innate immune pathways such as neutrophil response and coagulation, alongside suppression of adaptive immunity—a profile that persists even at the time of infection onset, suggesting a sustained predisposition to infection [9].

In this context, immune-inflammatory biomarkers capable of capturing both immune activation and suppression have attracted increasing attention. The systemic immune-inflammation index (SII), a novel composite biomarker calculated from platelet, neutrophil, and lymphocyte counts, reflects both innate immune activation and adaptive immune dysfunction [11]. Recent studies have shown that SII may correlate with postoperative infectious complications, including urinary tract infections and sepsis, underscoring its potential role in infection risk stratification [12, 13]. However, the association between SII and the risk of ICU-AI) remains insufficiently investigated. In this retrospective cohort study based on the MIMIC-IV database, we aimed to examine the association between SII and the subsequent risk of ICU-AI. Based on prior evidence, we hypothesized that elevated SII would be independently associated with an increased risk of ICU-AI.

## Methods

### Study design and setting

This retrospective cohort study was conducted in accordance with the Strengthening the Reporting of Observational Studies in Epidemiology guidelines [14]. The institutional review boards of the Massachusetts Institute of Technology and Beth Israel Deaconess Medical Center granted approval for the creation of the Medical Information Mart for Intensive Care (MIMIC)-IV database (version 3.1), a comprehensive clinical repository encompassing over 364,000 patients admitted from 2008 to 2022 at the Beth Israel Deaconess Medical Center [15]. One of the authors, Yulian Ren was given access to the database following completion of an online course and test (Certificate ID: 56915139). An informed consent waiver was secured due to the database comprising anonymized patient data, which lacks personally identifiable information.

### Inclusion and exclusion criteria

Patients were eligible if they met the following criteria: (1) first hospitalization with an initial ICU admission; (2) ICU stay longer than 48 h; (3) no suspected infection during the period from emergency department presentation to ICU admission, or during the first 48 h of ICU stay. Suspected infection was defined based on the concurrent presence of two events within a predefined temporal window: the administration of antibiotics (oral or intravenous) and the collection of body fluid cultures (e.g., blood, urine, or cerebrospinal fluid). If antibiotics were administered first, cultures must have been obtained within 24 h; if cultures were obtained first, antibiotics must have been initiated within 72 h. The complete list of antibiotic agents included in this definition is provided in Supplementary Table 1. The definition of suspected infection is derived from the standardized algorithm developed in the Sepsis-3 studies [16], which has been widely adopted in infection-related research using electronic health record databases due to its objectivity, reproducibility, and cross-study comparability [17, 18]. Exclusion criteria were as follows: (1) missing or zero values for any components of SII, including platelet, neutrophil, or lymphocyte counts within 24 h of ICU admission; (2) inconsistent timing of the first measurements of platelet, neutrophil, or lymphocyte counts; (3) biologically implausible or extreme outliers in key covariates.

### Exposure and outcomes

The Systemic Immune-Inflammation Index.

SII was calculated using the formula [11]:

$$\eqalign{ SII & = (platelet\,count \times neutrophil\,count) \cr & \div lymphocyte\,count \cr}$$

All parameters were obtained from the first values within 24 h after ICU admission. Although universal reference intervals for SII have not been formally established, previous studies have reported its distribution across various populations. In non-hospitalized populations, SII levels are generally below 1000 × 10⁹/L [19]. Among patients with sepsis, the median SII is approximately 1700 × 10⁹/L [12, 20].

### Outcome

The outcome was ICU-AI, defined as the first suspected infection occurring more than 48 h after ICU admission. Infection sites were determined according to the anatomical origin of the microbiological culture associated with the suspected infection event, regardless of whether the culture result was positive or negative. For patients with multiple culture tests, the earliest qualifying culture within the predefined time window was selected for each antibiotic event to define the infection episode.

### Follow-up

Follow-up began at ICU admission and continued for 28 days.

### Covariates

Clinical covariates were selected based on clinical relevance and prior literature related to HAIs [5, 21–25]. The variables included demographic characteristics, ICU admission details, vital signs, laboratory data, comorbidities, disease severity scores, and interventions prior to the onset of ICU-AI. All data were extracted using Navicat Premium (version 16.1.2) and Structured Query Language. Comorbidities were abstracted from ICD‑9 and ICD‑10 diagnostic codes documented in electronic health records. The specific codes used to define each comorbidity included in this study are provided in Supplementary Table 2. Vital signs were computed as average measurements taken over the first 24 h after the patient was admitted to the ICU. Laboratory values were gathered from the initial test findings available within the first day following ICU entry. Disease severity was quantified using SOFA, SAPS II, and Charlson comorbidity index within 24 h of ICU admission. Variables with more than 40% missing values were excluded from analysis. For variables with ≤ 40% missingness, multiple imputations by chained equations were applied under the assumption of missing at random. A sensitivity analysis comparing results before and after imputation was conducted to assess the robustness of findings (Supplementary Table 3).

### Competing risk

In this study, the development of ICU-AI was subject to competing risks, specifically ICU discharge and death, which could preclude observation of the primary outcome. We implemented two validated competing-risk methodologies: the proportional cause-specific hazard (CSH) model and the Fine-Gray subdistribution hazard model [26–28]. The CSH model considers competing events as censored observations and calculates the instantaneous infection risk among patients still at risk, thus rendering it appropriate for probing possible causal pathways between SII and infection onset. In contrast, the Fine-Gray model incorporates competing events into the risk set and estimates the cumulative incidence function, offering a comprehensive view of the overall impact of SII on infection risk over time.

### Statistical analysis

Baseline SII values were categorized into quartiles (Q1–Q4) for analysis. Descriptive statistics were used to summarize baseline characteristics. Continuous variables were expressed as medians with interquartile ranges (IQRs) and compared across SII quartiles using the Kruskal–Wallis test. Categorical variables were presented as counts and percentages and compared using the chi-square test or Fisher’s exact test, as appropriate. The association between SII and ICU-AI was evaluated using univariate and multivariate regression models. SII was analyzed as both a continuous variable (per 1,000-unit increase) and a categorical variable based on quartiles (Q1 as reference). Generalized variance inflation factors (GVIFs) were calculated to assess multicollinearity among covariates. We constructed three competing risk models: unadjusted model, partially adjusted model (adjusted for all covariates except SOFA and SAPS II), and fully adjusted model (adjusted for all covariates). The proportional hazards assumption for the CSH model was assessed using the Schoenfeld residual test. Cause-specific hazard ratios (CSHR) and subdistribution hazard ratios (SHR), along with their corresponding 95% confidence intervals (CIs), were reported to reflect results from both proportional cause-specific and Fine-Gray models, respectively. To illustrate the time-dependent distribution of ICU-AI risk across SII groups, cumulative cause-specific hazard curves derived from the CSH model and cumulative incidence function curves derived from the Fine-Gray subdistribution hazard model were plotted. Gray’s test was applied to assess differences in cumulative incidence across the SII quartiles. Subgroup analyses were conducted to explore potential heterogeneity of the association between SII and ICU-AI across predefined strata, including sex, age, race, diabetes, SOFA score, use of IMV and invasive line placement. Interaction terms were included in multivariable models to test for effect modification.

All analyses were performed using R version 4.2.2, which can be accessed from The R Foundation at http://www.R-project.org, and the Free Statistics software, version 2.1, available at http://www.clinicalscientists.cn/freestatistics. All tests were two-tailed, and a p-value < 0.05 was considered statistically significant.

### Sensitivity analysis

We performed three sensitivity analyses to assess the robustness of our findings. First, to evaluate the incremental predictive value of SII beyond established clinical variables, we compared Harrell’s C-index between CSH models with and without SII, thereby quantifying the contribution of SII to overall model discrimination. Second, given that WBC encompasses neutrophils and lymphocytes, we sought to examine whether inclusion of WBC could lead to overadjustment or distortion of the SII effect estimate. To this end, we refitted both competing risk models after excluding WBC while keeping all other covariates unchanged, and compared the results with those from the primary analysis. Third, to evaluate the potential impact of outcome misclassification, we redefined the primary outcome as culture-positive ICU-acquired infection, requiring a positive microbiological culture result for a suspected infection episode to be considered confirmed. All other inclusion and exclusion criteria, the competing risk modeling framework, and covariate adjustments remained identical to those in the primary analysis.

## Results

### Study population and baseline characteristics

**Fig. 1:**
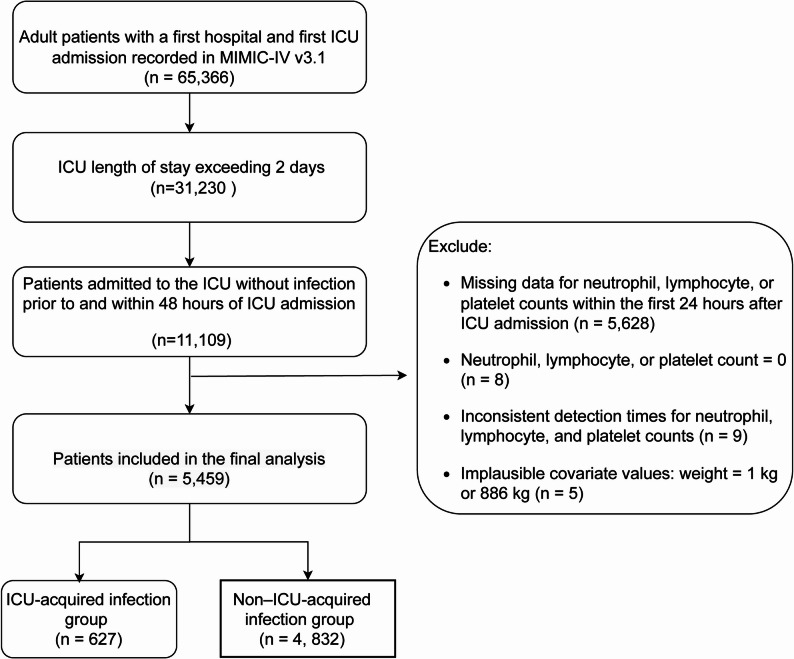
Flowchart of the study population. Abbreviations: MIMIC-IV v3.1 = Medical Information Mart for Intensive Care-IV database, version 3.1; ICU = intensive care unit

A total of 5,459 patients were included in the final analysis (Fig. 1). The cohort had a median age of 67.7 years (IQR 55.5–78.5) with 2,290 (41.9%) female participants. Baseline characteristics and clinical outcomes stratified by SII quartiles are presented in Table 1. The median SII level within 24 h of ICU admission for the entire cohort was 1125.3 × 10⁹/L (IQR 593.6–2201.3). During follow-up, 4,656 patients (85.3%) were discharged from the ICU without developing ICU-AI, 174 patients (3.2%) died before developing ICU-AI, and the remaining 2 patients (0.04%) experienced no event within the 28-day follow-up period. A total of 627 patients (11.5%) developed ICU-AI, and the incidence rates demonstrated a significant stepwise increase across ascending SII quartiles (*p* < 0.001). The median time from ICU admission to infection onset was 3.8 days (IQR, 2.8–5.1). The distribution of infection sites among ICU-AI cases is presented in Table 2. Bloodstream infections represented the most common site (35.1%, *n* = 220), followed by urinary tract (26.8%, *n* = 168) and respiratory tract infections (21.4%, *n* = 134).

**Table 1:** Demographics and characteristics of participants

Variables	Total (*n* = 5459)	Q1 (*n* = 1365)	Q2 (*n* = 1364)	Q3 (*n* = 1365)	Q4 (*n* = 1365)	*P*-value
SII, ×10⁹/L, Median (IQR)	1125.3 (593.6, 2201.3)	381.5 (268.9, 493.3)	823.5 (702.9, 970.6)	1520.3 (1304.2, 1815.9)	3422.6 (2713.7, 4839.8)	< 0.001
**Demographics**						
Female sex, n (%)	2290 (41.9)	535 (39.2)	544 (39.9)	567 (41.5)	644 (47.2)	< 0.001
Age, year, Median (IQR)	67.7 (55.5, 78.5)	68.0 (56.7, 78.0)	68.6 (56.9, 79.0)	67.3 (55.2, 78.8)	66.6 (53.2, 78.1)	0.026
Race, White, n (%)	3354 (61.4)	839 (61.5)	848 (62.2)	825 (60.4)	842 (61.7)	0.821
BMI, kg/m², median (IQR)	27.7 (24.0, 32.1)	27.7 (24.3, 32.1)	28.3 (24.5, 32.4)	27.7 (24.1, 32.0)	27.0 (23.2, 31.7)	< 0.001
History of tobacco use, n (%)	2038 (37.3)	539 (39.5)	538 (39.4)	492 (36.0)	469 (34.4)	0.01
Admission type, n(%)						
Scheduled Surgical	101 (1.9)	54 (4.0)	24 (1.8)	17 (1.2)	6 (0.4)	
Unscheduled Surgical	2119 (38.8)	628 (46.0)	552 (40.5)	488 (35.8)	451 (33.0)	
Medical	3239 (59.3)	683 (50.0)	788 (57.8)	860 (63.0)	908 (66.5)	
ICU type, n (%)						< 0.001
Cardiac ICU	1893 (34.7)	643 (47.1)	500 (36.7)	421 (30.8)	329 (24.1)	
General ICU	1863 (34.1)	371 (27.2)	417 (30.6)	488 (35.8)	587 (43.0)	
Neuro ICU	1214 (22.2)	278 (20.4)	347 (25.4)	322 (23.6)	267 (19.6)	
Trauma ICU	489 (9.0)	73 (5.3)	100 (7.3)	134 (9.8)	182 (13.3)	
**Treatment before infection**						
Days on IMV, days, Median (IQR)	1.4 (0.1, 2.5)	1.3 (0.0, 2.4)	1.2 (0.0, 2.4)	1.4 (0.1, 2.6)	1.5 (0.3, 2.7)	< 0.001
Days on invasive line, days, edian (IQR)	0.0 (0.0, 3.0)	0.0 (0.0, 3.2)	0.0 (0.0, 2.9)	0.0 (0.0, 3.0)	0.0 (0.0, 3.1)	0.147
Days on RRT, days, Median (IQR)	0.0 (0.0, 0.0)	0.0 (0.0, 0.0)	0.0 (0.0, 0.0)	0.0 (0.0, 0.0)	0.0 (0.0, 0.0)	0.273
**Vital signs**						
Heart rate, bpm, Median (IQR)	79.3 (70.8, 90.0)	78.0 (69.9, 86.7)	78.5 (70.5, 88.0)	80.0 (71.2, 91.0)	82.0 (72.0, 94.2)	< 0.001
MBP, mmHg, Median (IQR)	80.5 (73.8, 89.1)	79.2 (73.0, 88.5)	80.4 (73.5, 89.5)	81.2 (74.0, 89.9)	81.2 (74.5, 88.5)	0.004
Respiratory rate, bpm, Median (IQR)	18.2 (16.4, 20.3)	17.7 (16.1, 19.5)	18.2 (16.5, 20.1)	18.4 (16.6, 20.7)	18.7 (16.6, 21.2)	< 0.001
Temperature,℃, Median (IQR)	36.8 (36.6, 37.0)	36.7 (36.6, 36.9)	36.8 (36.6, 37.0)	36.8 (36.6, 37.0)	36.8 (36.6, 37.1)	< 0.001
Spo2, %, Median (IQR)	97.0 (95.6, 98.4)	97.4 (95.9, 98.5)	97.0 (95.8, 98.2)	96.9 (95.4, 98.3)	96.9 (95.4, 98.4)	< 0.001
Blood glucose, mg/dL, Median (IQR)	127.2 (109.2, 149.0)	124.2 (107.6, 141.9)	126.0 (106.0, 146.2)	127.5 (109.7, 151.6)	132.0 (113.5, 159.0)	< 0.001
**Comorbidity**						
AIDS, n (%)	11 (0.2)	7 (0.5)	2 (0.1)	1 (0.1)	1 (0.1)	0.038
COVID-19, n (%)	120 (2.2)	15 (1.1)	25 (1.8)	29 (2.1)	51 (3.7)	< 0.001
Diabetes, n(%)	1501 (27.5)	380 (27.8)	395 (29)	379 (27.8)	347 (25.4)	0.209
Cerebrovascular disease, n(%)	1635 (30.0)	352 (25.8)	413 (30.3)	457 (33.5)	413 (30.3)	< 0.001
Metastatic solid tumor, n(%)	232 (4.2)	27 (2)	39 (2.9)	67 (4.9)	99 (7.3)	< 0.001
Congestive heart failure, n (%)	1390 (25.5)	320 (23.4)	346 (25.4)	374 (27.4)	350 (25.6)	0.129
Chronic pulmonary disease, n(%)	1111 (20.4)	269 (19.7)	257 (18.8)	268 (19.6)	317 (23.2)	0.022
Rheumatic disease, n (%)	158 (2.9)	38 (2.8)	35 (2.6)	52 (3.8)	33 (2.4)	0.124
Renal disease, n (%)	986 (18.1)	259 (19.0)	249 (18.3)	239 (17.5)	239 (17.5)	0.714
Liver disease, n (%)	378 (6.9)	129 (9.5)	82 (6.0)	71 (5.2)	96 (7.0)	< 0.001
**Scoring system**						
Sofa score, Median (IQR)	3.0 (2.0, 5.0)	4.0 (2.0, 6.0)	3.0 (2.0, 5.0)	3.0 (1.0, 5.0)	3.0 (1.0, 5.0)	< 0.001
SAPS II, Median (IQR)	32.0 (25.0, 40.0)	33.0 (25.0, 40.0)	33.0 (26.0, 40.0)	31.0 (24.0, 39.0)	32.0 (24.0, 40.0)	0.124
CCI, Median (IQR)	5.0 (3.0, 7.0)	4.0 (3.0, 6.0)	5.0 (3.0, 7.0)	5.0 (3.0, 7.0)	5.0 (2.0, 7.0)	0.74
**Laboratory parameters**						
RBC count, ×10¹²/L, Median (IQR)	4.0 (3.3, 4.6)	3.6 (2.9, 4.3)	4.0 (3.3, 4.5)	4.1 (3.5, 4.6)	4.1 (3.5, 4.6)	< 0.001
WBC count, ×10⁹/L, Median (IQR)	10.2 (7.7, 13.6)	7.9 (6.0, 10.3)	9.4 (7.4, 12.3)	10.9 (8.7, 13.5)	13.4 (10.4, 17.1)	< 0.001
BUN, mg/dL, Median (IQR)	18.0 (13.0, 26.0)	17.0 (12.0, 23.0)	17.0 (12.0, 25.0)	18.0 (13.0, 27.0)	19.0 (13.0, 30.0)	< 0.001
Creatinine, mg/dL, Median (IQR)	0.9 (0.7, 1.3)	0.9 (0.7, 1.2)	0.9 (0.7, 1.2)	1.0 (0.8, 1.3)	1.0 (0.7, 1.4)	0.005
PT, S, Median (IQR)	13.0 (11.8, 15.5)	13.6 (12.0, 16.5)	13.0 (11.8, 15.6)	12.8 (11.7, 15.2)	12.7 (11.8, 14.8)	< 0.001
PTT, S, Median (IQR)	29.2 (26.1, 33.9)	30.3 (27.0, 35.5)	29.3 (26.3, 33.7)	28.7 (25.7, 33.1)	28.5 (25.6, 33.5)	< 0.001
**Outcome**						
ICU-acquired infection, n (%)	627 (11.5)	101 (7.4)	128 (9.4)	168 (12.3)	230 (16.8)	< 0.001
Interval from ICU admission to infection, days, Median (IQR)	3.8 (2.8, 5.1)	3.6 (2.8, 5.3)	3.6 (2.9, 5.1)	3.8 (2.9, 5.6)	3.8 (2.7, 4.9)	0.604
LOS-H, days, Median (IQR)	7.6 (4.9, 12.5)	6.9 (4.7, 11.4)	7.0 (4.8, 11.4)	7.7 (5.1, 12.7)	8.4 (5.1, 14.7)	< 0.001
LOS-ICU, days, Median (IQR)	3.3 (2.5, 5.3)	3.1 (2.4, 4.6)	3.3 (2.5, 5.1)	3.4 (2.6, 5.7)	3.7 (2.6, 6.2)	< 0.001

Abbreviations: SII, systemic immune-inflammation index; IQR, interquartile range; BMI, body mass index; ICU, intensive care unit; IMV, invasive mechanical ventilation; RRT, renal replacement therapy; MBP, mean blood pressure; SpO2, peripheral blood oxygen saturation; AIDS: acquired immune deficiency syndrome; COVID-19, coronavirus disease 2019; SOFA, sequential organ failure assessment; SAPSⅡ, Simplified Acute Physiology Score Ⅱ; CCI, Charlson comorbidity index; RBC, red blood cell; WBC, white blood cell; BUN, blood urea nitrogen; PT, prothrombin time; PTT, partial thromboplastin time, LOS-H, the length of stay in hospital; LOS-ICU, the length of stay in ICU

**Table 2:** Infection sites among ICU-AI patients stratified by SII quartiles

	Total (*n* = 627)	Q1 (*n* = 101)	Q2 (*n* = 128)	Q3 (*n* = 168)	Q4 (*n* = 230)	*P*-value
Infection site, *n* (%)						0.345
Bloodstream	220 (35.1)	33 (32.7)	44 (34.4)	65 (38.7)	78 (33.9)	
Urinary tract	168 (26.8)	35 (34.7)	32 (25.0)	45 (26.8)	56 (24.3)	
Respiratory tract	134 (21.4)	20 (19.8)	28 (21.9)	38 (22.6)	48 (20.9)	
Other sites	105 (16.7)	13 (12.9)	24 (18.8)	20 (11.9)	48 (20.9)	

### Association between SII and ICU-AI

Prior to multivariable modeling, multicollinearity was assessed using generalized variance inflation factors. Mean blood pressure, the Charlson Comorbidity Index, and red blood cell count were excluded because their GVIF^(1/(2×Df)) exceeded 2.5. All other covariates were retained with acceptable collinearity metrics (Supplementary Table 4).

Table 3 presents the estimates and 95% confidence intervals of the CSHR and SHR for ICU-AI associated with increasing SII levels. When modeled as a continuous variable (per 1,000-unit increase), SII was independently associated with a higher hazard of infection (adjusted CSHR = 1.06; 95% CI: 1.03–1.08; *p* < 0.001) in fully adjusted model. When analyzed by quartiles, compared with patients in the lowest quartile, those in the third and fourth quartiles had significantly higher risks of ICU-AI, with adjusted CSHR of 1.44 (95% CI: 1.11–1.86; *p* = 0.006) and 1.76 (95% CI: 1.37–2.26; *p* < 0.001), respectively (p for trend < 0.001). These results were consistent with those from the Fine-Gray subdistribution hazard model (adjusted SHR for Q4, 1.96; 95% CI, 1.54–2.49; *p* < 0.001). The results of the proportional hazards assumption test for the CSH model are presented in Supplementary Table 5. The test for SII was not significant (*p* = 0.368), indicating that the effect of SII on ICU-AI risk remained constant over the 28-day follow-up period and satisfied the model assumption. Although the global test was significant (*p* < 0.001) due to time-varying effects of several covariates (e.g., mechanical ventilation duration, age), this does not compromise the validity of the effect estimate for the primary exposure.

**Fig. 2:**
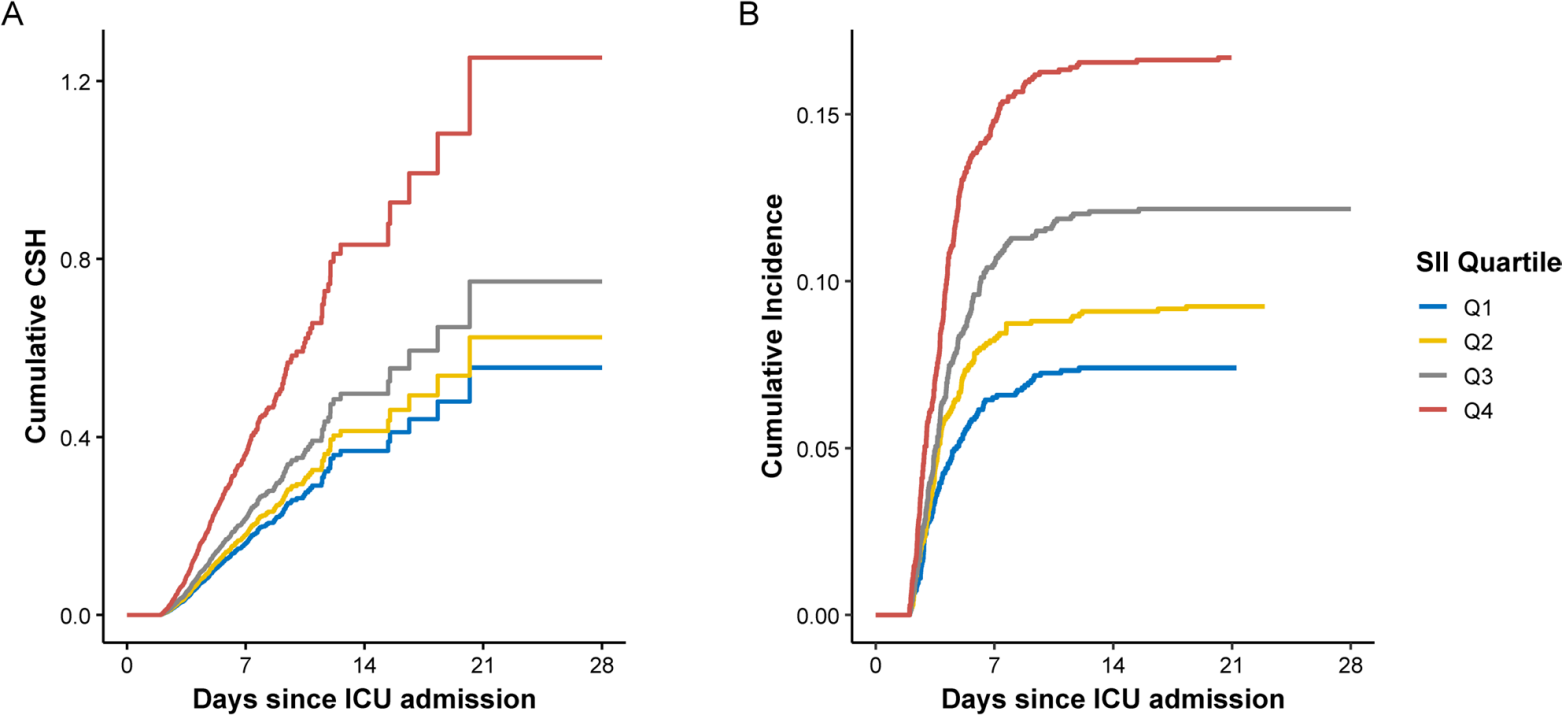
Time -to-event risk of ICU-AI across SII quartiles. **(A)** Cumulative cause-specific hazard curves for ICU-AI by SII quartile, based on proportional cause-specific hazard model. **(B)** Cumulative incidence function curves from Fine-Gray models comparing the probability of ICU-AI across SII quartiles in the presence of competing risks (ICU discharge and death). Abbreviations: SII, systemic immune-inflammation index; ICU, intensive care unit; CSH, cause-specific hazard

**Fig. 3:**
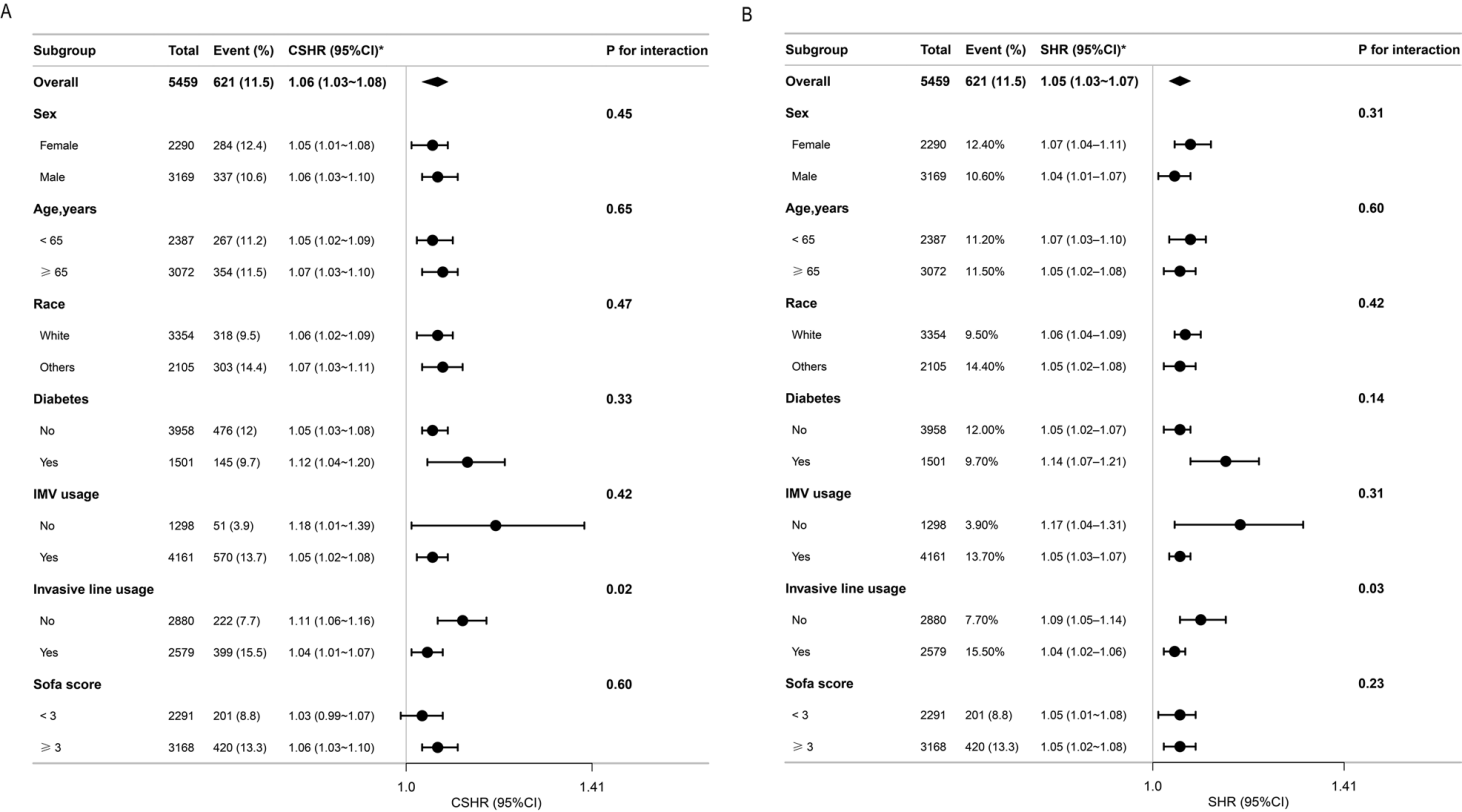
Subgroup analysis of the association between SII and ICU-AI. *HR (95%CI) were obtained from regression models adjusted for variables in fully adjusted model as shown in Table 3. Abbreviations: SII, systemic immune-inflammation index; CSHR, cause-specific hazard ratio; SHR, subdistribution hazard ratio; CI, confidence interval; ICU, intensive care unit; IMV, invasive mechanical ventilation; SOFA, sequential organ failure assessment

Figure 2 displays the cumulative CSH curves and cumulative incidence functions for ICU-AI across SII quartiles. Both curves demonstrated a clear dose–response relationship (*p* < 0.001), with patients in higher SII quartiles exhibiting significantly higher cumulative risk over time. The corresponding 28-day cumulative incidence of ICU-AI increased progressively across SII quartiles: 7.4% (95% CI 6.0–8.8) in Q1, 9.2% (95% CI 7.7–10.8) in Q2, 12.2% (95% CI 10.4–13.9) in Q3, and 16.7% (95% CI 14.7–18.7) in Q4. Stratified analyses confirmed that the association between SII and ICU-AI remained robust across all subgroups (Fig. 3.

**Table 3:** Relationship between SII and ICU-AI in different models

Variable	Proportional cause-specific hazard model			Fine-Gray subdistribution hazard model		
	Crude CSHR (95%CI) (*P*-value)	Partially Adjusted CSHR * (95%CI) (*P*-value)	Fully Adjusted CSHR ** (95%CI) (*P*-value)	Crude SHR (95%CI)(*P*-value)	Partially Adjusted SHR* (95%CI) (*P*-value)	Fully Adjusted SHR ** (95%CI) (*P*-value)
SII, ×10⁹/L (Pre 1000-unit)	1.06 (1.04 ~ 1.09) (< 0.001)	1.05 (1.03–1.08) (< 0.001)	1.06 (1.03 ~ 1.08) (< 0.001)	1.05 (1.03 ~ 1.08) (< 0.001)	1.05 (1.03–1.07) (< 0.001	1.05 (1.03 ~ 1.07) (< 0.001)
SII quartile, ×10^9^/L						
Q1 (≤ 594)	1.0 (reference)	1.0 (reference)	1.0 (reference)	1.0 (reference)	1.0 (reference)	1.0 (reference)
Q2 (595–1124)	1.15 (0.89 ~ 1.50) (0.285)	1.12 (0.86–1.45) (0.421)	1.19 (0.91 ~ 1.56) (0.198)	1.26 (0.97 ~ 1.64) (0.082)	1.22 (0.94–1.59) (0.130)	1.28 (0.99 ~ 1.66) (0.064)
Q3 (1125–2201)	1.35 (1.06 ~ 1.73) (0.016)	1.26 (0.98–1.62) (0.074)	1.44 (1.11 ~ 1.86) (0.006)	1.68 (1.31 ~ 2.15) (< 0.001)	1.39 (1.08–1.78) (0.011)	1.51 (1.17 ~ 1.93)(< 0.001)
Q4 (≥ 2202)	1.84 (1.45 ~ 2.32) (< 0.001)	1.60 (1.26–2.04) (< 0.001)	1.76 (1.37 ~ 2.26) (< 0.001)	2.38 (1.88 ~ 3.00) (< 0.001)	1.86 (1.46–2.36) (0.000)	1.96 (1.54 ~ 2.49) (< 0.001)
P for trend	< 0.001	< 0.001	< 0.001	< 0.001	< 0.001	< 0.001

*adjusted for age, race, sex, BMI, history of tobacco use, ICU type, admission type, treatment before infection (days on IMV, days on invasive line, days on RRT), vital signs (heart rate, respiratory rate, temperature, Spo2 and blood glucose), comorbidity (AIDS, COVID-19, diabetes, cerebrovascular disease, metastatic solid tumor, congestive heart failure, chronic pulmonary disease, rheumatic disease, renal disease and liver disease), laboratory parameters (WBC count, BUN, creatinine, PT and PTT)

**adjusted for variables in partially adjusted model + SOFA score, SAPS Ⅱ

Abbreviations: SII, systemic immune-inflammation index; ICU, intensive care unit; CSHR, cause-specific hazard ratio; SHR, subdistribution hazard ratio; CI, confidence interval

### Sensitivity analysis results

First, to evaluate the incremental predictive value of SII beyond established clinical variables, we compared Harrell’s C-index between CSH models with and without SII. The C-index for the base model (without SII) was 0.656 (SE = 0.013), which increased to 0.665 (SE = 0.013) after adding SII, representing an absolute improvement of 0.008. Second, given that WBC overlaps with the components of SII, we refitted the models after excluding WBC while keeping all other covariates unchanged. The effect estimates for SII per 1000 × 10⁹/L increase were nearly identical to those from the primary analysis (Supplementary Table 6). Third, when the outcome was restricted to culture-positive suspected infection, a total of 621 patients (11.4%) met the criteria for ICU-AI. The effect estimates for SII remained consistent with those observed in the primary analysis (Supplementary Table 7).

## Discussion

In this large retrospective cohort study involving 5,459 ICU patients without evidence of baseline infection, we found that elevated SII levels at ICU admission were significantly associated with an increased risk of developing suspected secondary infection. This study adds to the limited body of evidence linking SII to the risk of HAIs. To our knowledge, it is the first investigation to examine the association between SII and ICU-AI while appropriately accounting for competing risks. In our cohort, patients with elevated SII were observed to have significantly longer ICU and hospital stays, resulting in extended exposure to infection risk. However, CSH model adjusts for this potential time-dependent confounding by estimating the instantaneous hazard of infection among individuals who remain at risk. A CSHR greater than 1 thus reflects an elevated instantaneous rate of suspected infection among those still at risk in the elevated SII group. Additionally, the Fine-Gray subdistribution hazard ratio greater than 1 indicates that, even when accounting for the likelihood of early ICU discharge or death, patients with elevated SII still experienced a significantly higher cumulative incidence of ICU-AI over time.

Several previous studies have explored the potential of SII as an early biomarker for infection risk in various patient populations and clinical settings. For example, in a retrospective analysis of patients undergoing retrograde intrarenal surgery, elevated preoperative SII was associated with postoperative fever and urinary tract infections, even in those with sterile preoperative urine cultures [13]. Similarly, a pediatric study involving infants with fever without a source found that SII demonstrated strong discriminative ability in identifying serious bacterial infections [29]. These findings are consistent with our results and suggest that elevated SII may reflect a subclinical inflammatory state that precedes clinically apparent infection. While both prior studies underscored SII as a potential biomarker for infection risk, our work extends this evidence to a critically ill adults, a population characterized by marked immune dysregulation and increased vulnerability to infection. SII quartiles provide an intuitive framework for risk stratification: among infection-naïve ICU patients, the cumulative incidence of suspected ICU-acquired infection rose progressively with increasing baseline SII. This graded risk pattern offers a quantifiable reference for understanding the relative burden of suspected infection across different levels of inflammatory predisposition. However, whether this gradient can be translated into improved patient outcomes through specific clinical strategies remains to be established in prospective interventional studies. Specifically, hypotheses warranting future investigation include: (1) whether SII-stratified infection surveillance protocols can enhance the early detection of suspected ICU-acquired infections; (2) whether SII can be effectively integrated into electronic health record-based risk-stratification tools to support clinical decision-making; and (3) whether combining SII with other biomarkers, such as procalcitonin or C-reactive protein, can achieve more precise risk discrimination than that afforded by existing clinical variables alone.

It is important to note that SOFA and SAPS II scores capture downstream physiologic consequences of systemic inflammation, and thus may partially overlap with the inflammatory pathways reflected by SII. Consequently, the fully adjusted estimates presented in this study should be interpreted as demonstrating the robustness of the association after accounting for overall illness severity, rather than as evidence of a biological effect that is entirely independent of the inflammatory cascade. In other words, SII remains associated with ICU-acquired infection even when controlling for organ dysfunction, but this does not preclude the possibility that SII and severity scores share common upstream determinants.

It is also worth reflecting on the time-varying effects observed for several covariates in the proportional hazards assessment. Although baseline SII satisfied the proportional hazards assumption, multiple other variables exhibited statistically significant time-varying effects, including duration of IMV, days with invasive lines, age, and cerebrovascular disease, among others. In critically ill patients, whose clinical status and therapeutic exposures evolve rapidly over the course of an ICU stay, such temporal dynamism in infection risk is clinically plausible. For example, ventilator-associated pneumonia is among the most common ICU-AI. Its incidence has been reported to peak between days 3 and 7 of IMV and to decline thereafter, a pattern attributable in part to heightened clinical vigilance and improved adherence to preventive bundles [30]. Similarly, rates of central line-associated bloodstream infection accumulate non-linearly with catheter dwell time, with the incremental risk being highest during the early catheterization period [31, 32]. Cerebrovascular disease is also associated with a distinct temporal pattern of nosocomial infection. Post-stroke pneumonia has been consistently shown to peak within the first week following stroke onset [33]. While these time-varying effects do not compromise the validity of the estimated hazard ratio for a time-constant exposure such as baseline SII, they underscore the complex, time-dependent nature of infection risk in critically ill patients.

This study has three major strengths. First, SII is derived from routine complete blood count parameters, making it a highly accessible biomarker that requires no additional testing. We evaluated its incremental predictive value in a model that included over 30 clinical variables. The addition of SII improved the C-index from 0.656 to 0.665. Although this absolute improvement in discrimination is statistically consistent with a positive contribution, it does not meet conventional thresholds for clinically meaningful predictive enrichment. We therefore interpret this finding cautiously: SII may provide modestly complementary risk information beyond established clinical variables, but the magnitude of improvement is small, and its clinical utility requires further evaluation in decision-analytic frameworks. Coupled with its low cost and routine availability, SII may serve as an accessible supplementary indicator for early risk awareness, rather than as a stand-alone predictive tool. Second, our application of two established competing risk models effectively addresses biases from competing events, providing more reliable risk estimates than traditional survival analyses. Third, by excluding patients with suspected infections at ICU admission or within 48 h, this study characterizes the relationship between baseline SII and the subsequent development of suspected infection in patients initially free of infection. This study population, while selected, allows for a cleaner assessment of the temporal sequence between inflammatory status at admission and subsequent infection events. This distinction supports the potential utility of SII as an early risk-stratification tool for infection-naïve ICU patients. Specifically, the SII value could help identify a subgroup of patients who, despite having no evidence of infection upon ICU arrival, may warrant heightened surveillance or earlier diagnostic evaluation for nosocomial infections. Such a targeted approach could complement existing infection control practices without imposing additional burdens on all admissions. It is critical to emphasize, however, that these implications apply exclusively to patients without suspected infection at ICU entry; our findings do not validate SII as a universal risk marker for the broader ICU population, particularly those admitted with sepsis or established infection—a population that is inherently at greater risk for secondary infections. The immune-inflammatory status in the latter population is more complex: on one hand, infection itself can increase platelet and neutrophil counts, directly elevating SII levels [34, 35]; on the other hand, some patients may develop immunoparalysis, increasing the risk of secondary infections [7, 8]. As a result, the predictive value of SII in this context may be bidirectional, with both extremely high and low levels potentially associated with adverse outcomes.

Nonetheless, this study also has limitations. First, as a retrospective single-center study, it is inherently subject to biases related to data availability, unmeasured confounders, and potential selection effects. Causal relationships between SII and ICU-AI cannot be inferred from this study design. Moreover, being limited to one center, the generalizability of our findings may be restricted due to variations in healthcare delivery, patient populations, or infection control practices. Second, our covariate adjustment was limited to variables available in the electronic health record. Important factors such as adherence to hand hygiene, or environmental sanitation could not be assessed, which may have influenced infection risk. Third, the operational definition of ICU-acquired infection in our study, which relied on antibiotic initiation and microbiological sampling rather than prospective clinical adjudication, might potentially introduce some degree of misclassification bias. False positives may occur when antibiotics are administered for non-infectious indications (e.g., prophylaxis) alongside culture collection, while false negatives may occur when true infections are missed due to delayed or omitted cultures. Specifically, clinicians may be more likely to initiate antimicrobial therapy and order cultures in patients with elevated SII due to their heightened inflammatory presentation, independent of true infection status. This differential detection bias could inflate the observed association. To explore this possibility, we performed a sensitivity analysis restricting the outcome to culture-positive ICU-acquired infections. The association remained robust, suggesting that differential ascertainment bias is unlikely to explain the observed relationship. Notwithstanding these constraints, our case identification approach demonstrates clinical validity through its alignment with the clinical suspicion component of Sepsis-3 infection criteria. The standardized methodology employed enhances reproducibility across diverse ICU settings while reducing interobserver variability in infection surveillance - an essential feature for large-scale database research.

## Conclusion

The present study demonstrates that an elevated SII at ICU admission is an independent risk factor for the development of suspected secondary infection in infection-naïve critically ill patients. This finding may facilitate early identification of high-risk individuals; however, future prospective studies are warranted to validate the prognostic value of SII and to evaluate its incorporation into existing risk-stratification models.

## Electronic Supplementary Material

Below is the link to the electronic supplementary material.

Supplementary Material 1

## Data Availability

The data that supports the findings of this study are available in MIMIC-IV (Medical Information Mart for Intensive Care) at 10.13026/kpb9-mt58, reference number 10.13026/kpb9-mt58. These data were derived from the following resources available in the public domain: MIMIC-IV Clinical Database, Version 3.1, .

## Abbreviations

CI: Confidence interval
CSHR: Cause-specific hazard ratio
GVIF: Generalized variance inflation factor
HAI: Healthcare-associated infection
ICU: Intensive care unit
ICU-AI: ICU-acquired infection
IMV: Invasive mechanical ventilation
IQR: Interquartile range
LOS-H: The length of stay in hospital
LOS-ICU: The length of stay in ICU
MIMIC: Medical Information Mart for Intensive Care
NET: Neutrophil extracellular trap
PNCs: Platelet-neutrophil complexes
RRT: Renal replacement therapy
SHR: Subdistribution hazard ratio
SII: Systemic immune-inflammation index
SOFA: Sequential organ failure assessment
WBC: White blood cell
