# Supplementary material for: Association between systemic immune-inflammation index and ICU-acquired infection in critically Ill patients without infection: a competing risks analysis from a retrospective cohort
Source: BMC Infect Dis. 2026 May 18;26:1311. doi: 10.1186/s12879-026-13568-0 (PMC13366857; doi:10.1186/s12879-026-13568-0)
Supplement: Supplementary file 1 — Supplementary Material 1 [file 12879_2026_13568_MOESM1_ESM.pdf]

Supplementary Material

Supplementary Table 1. Antibiotics used to define suspected infection 1

Supplementary Table 2. ICD-9 and ICD-10 Codes for Comorbidities 3

Supplementary Table 3. Sensitivity analysis for multiple imputation of missing variables 3

Supplementary Table 4. Assessment of multicollinearity using generalized variance inflation factor prior to multivariable modeling 4

Supplementary Table 5. Schoenfeld residual test for proportional hazards assumption in the fully adjusted cause- specific hazards model 7

Supplementary Table 6. Full Model and Sensitivity Analysis (Excluding WBC) for the Association between SII and ICU-AI 8

Supplementary Table 7. Sensitivity analysis comparing the association between SII and culture-positive ICU-acquired infection 10

Supplementary Table 1. Antibiotics used to define suspected infection

Antibiotic Class	Drug Name
Penicillins	Amoxicillin, Ampicillin, Augmentin, Clavulanate, Penicillin, Nafcillin, Oxacillin, Dicloxacillin, Methicillin, Piperacillin, Tazobactam, Timentin, Unasyn, Zosyn
Cephalosporins	Cefazolin, Cefoxitin, Ceftazidime, Cefaclor, Cefadroxil, Cefdinir, Cefditoren, Cefepime, Cefotetan, Cefotaxime, Ceftaroline, Cefpodoxime, Cefprozil, Ceftibuten, Ceftriaxone, Cefuroxime, Cephalexin, Cephalothin, Cephapirin
Carbapenems	Meropenem,Imipenem, Ertapenem, Doripenem, Panipenem, Biapenem, Tebipenem

Antibiotic Class	Drug Name
Monobactams	Aztreonam
Tetracyclines	Doxycycline, Minocycline, Tetracycline
Macrolides	Azithromycin, Clarithromycin, Erythromycin, Dirithromycin
Fluoroquinolones	Ciprofloxacin, Levofloxacin, Moxifloxacin, Ofloxacin, Norfloxacin
Aminoglycosides	Amikacin, Gentamicin, Kanamycin, Neomycin, Streptomycin, Tobramycin
Sulfonamides/Trimethoprim	Sulfamethoxazole/Trimethoprim, Sulfadiazine, Sulfisoxazole, Trimethoprim
Lincosamides	Clindamycin, Lincomycin
Glycopeptides	Vancomycin
Oxazolidinones	Linezolid
Lipopeptides	Daptomycin
Nitroimidazoles	Metronidazole
Other Antibiotics	Chloramphenicol, Nitrofurantoin, Rifampin, Mupirocin, Fosfomycin, Quinupristin/Dalfopristin
Antifungals	Amphotericin B, Anidulafungin

Notes: Only systemic antibiotics (oral or intravenous) were considered; topical, ophthalmic, and otic formulations were excluded.

Supplementary Table 2. ICD-9 and ICD-10 Codes for Comorbidities

Comorbidity	ICD-9-CM Codes	ICD-10-CM Codes
AIDS	042, 043, 044	B20, B21, B22, B24
COVID-19	—	U07.1, J12.82
Diabetes mellitus	2500–2503, 2508, 2509<br><br>2504–2507	E100, E10l, E106, E108, E109, E110, E111, E116, E118, E119, E120, E121, E126, E128, E129, E130, E131, E136, E138, E139, E140, E141, E146, E148, E149<br>E102, E103, E104, E105, E107, E112, E113, E114, E115, E117, E122, E123, E124, E125, E127, E132, E133, E134, E135, E137, E142, E143, E144, E145, E147
Cerebrovascular disease	430–438, 36234	G45, G46, I60–I69, H340
Metastatic solid tumor	196, 197, 198, 199	C77, C78, C79, C80
Congestive heart failure	428, 39891, 40201, 40211, 40291, 40401, 40403, 40411, 40413, 40491, 40493, 4254–4259	I43, I50, I099, I110, I130, I132, I255, I420, I425, I426, I427, I428, I429, P290
Chronic pulmonary disease	490–505, 4168, 4169, 5064, 5081, 5088	J40–J47, J60–J67, I278, I279, J684, J701, J703
Rheumatic disease	725, 4465, 7100, 7101, 7102, 7103, 7104, 7140, 7141, 7142, 7148	M05, M06, M32, M33, M34, M315, M351, M353, M360
Renal disease	582, 585, 586, V56, 5880, V420, V451, 5830–5837, 40301, 40311, 40391, 40402, 40403, 40412, 40413, 40492, 40493	N18, N19, I120, I131, N032–N037, N052–N057, N250, Z490, Z491, Z492, Z940, Z992
Liver disease	570, 571, 0706, 0709, 5733, 5734, 5738, 5739, V427, 07022, 07023, 07032, 07033, 07044, 07054 (mild)<br>4560, 4561, 4562, 5722–5728 (severe)	B18, K73, K74, K700–K703, K709, K713–K715, K717, K760, K762–K764, K768, K769, Z944 (mild)<br>I850, I859, I864, I982, K704, K711, K721, K729, K765–K767 (severe)

Supplementary Table 3. Sensitivity analysis for multiple imputation of missing variables

Variable	Before imputation	After imputation	P-value*	Miss percentage (%)
Height,cm,Median (IQR)	170.0 (163.0, 178.0)	170.0 (163.0, 178.0)	0.79	33.54
Weight,kg,Median (IQR)	79.3 (66.6, 94.1)	79.3 (66.6, 94.1)	0.99	0.37
Heart rate, bpm, Median (IQR)	79.3 (70.8, 90.0)	79.3 (70.8, 90.0)	0.98	0.15
MBP,mmHg, Median (IQR)	80.5 (73.8, 89.1)	80.5 (73.8, 89.1)	0.94	0.15
Respiratory rate, bpm, Median (IQR)	18.2 (16.4, 20.3)	18.2 (16.4, 20.3)	0.95	0.29
Temperature,℃, Median (IQR)	36.8 (36.6, 37.0)	36.8 (36.6, 37.0)	0.8	2.12
Spo2, %, Median (IQR)	97.0 (95.7, 98.4)	97.0 (95.6, 98.4)	0.99	0.15
Blood glucose, mg/dL, Median (IQR)	127.2 (109.3, 149.0)	127.2 (109.2, 149.0)	0.97	0.66
BUN, mg/dL, Median (IQR)	18.0 (13.0, 26.0)	18.0 (13.0, 26.0)	0.98	1.19
Creatinine, mg/dL, Median (IQR)	0.9 (0.7, 1.3)	0.9 (0.7, 1.3)	0.84	5.57
PT, S, Median (IQR)	12.9 (11.8, 15.5)	13.0 (11.8, 15.5)	0.61	4.56
PTT, S, Median (IQR)	29.2 (26.1, 33.8)	29.2 (26.1, 33.9)	0.75	4.8
RBC count, 1012/L, Median (IQR)	4.0 (3.3, 4.6)	4.0 (3.3, 4.6)	0.99	0.05

*Two-tailed p value based on Mann-Whitney U test.

Abbreviations: IQR, interquartile range; MBP, mean blood pressu; SpO2, peripheral blood oxygen saturation; BUN, blood urea nitrogen; PT, prothrombin time; PTT, partial thromboplastin time; RBC, red blood cell

Supplementary Table 4. Assessment of multicollinearity using generalized variance inflation factor prior to multivariable modeling

Variables	GVIF	DF	GVIF^(1/(2*Df))*	Colinearity
Sex	1.19	1	1.09	No
Age	4.79	1	2.19	No
Race	1.15	1	1.07	No
BMI	1.28	1	1.13	No
History of tobacco use	1.11	1	1.05	No
Admission type	1.33	1	1.15	No
ICU type	1.36	1	1.17	No
Days on IMV	1.91	1	1.38	No
Days on invasive line	4.08	1	2.02	No
Days on RRT	2.46	1	1.57	No
Heart rate	1.83	1	1.35	No
MBP	22.96	1	4.79	Yes
Respiratory rate	1.42	1	1.19	No
Temperature	1.24	1	1.11	No
Spo2	1.4	1	1.18	No
Blood glucose	1.01	1	1	No
AIDS	1.1	1	1.05	No
COVID-19	1.23	1	1.11	No
Diabetes	1.66	1	1.29	No
Cerebrovascular disease	1.75	1	1.32	No
Metastatic solid tumor	2.57	1	1.6	No
Congestive heart failure	1.73	1	1.31	No
Chronic pulmonary disease	1.35	1	1.16	No
Rheumatic disease	1.06	1	1.03	No
Renal disease	2.13	1	1.46	No
Liver disease	1.35	1	1.16	No

Sofa score	2.68	1	1.64	No
SAPS II	5.26	1	2.30	No
CCI	8.17	1	2.86	Yes
RBC count	8.05	1	2.84	Yes
WBC count	1.09	1	1.05	No
BUN	2.94	1	1.71	No
Creatinine	2.49	1	1.58	No
PT	1.15	1	1.07	No
PTT	1.14	1	1.07	No

*GVIF raised to the power of 1/(2*Df) greater than 2.5 was considered indicative of collinearity.

Abbreviations: GVIF, generalized variance inflation factor; DF, degree of freedom; BMI, body mass index; ICU, intensive care unit; IMV, invasive mechanical ventilation; RRT, renal replacement therapy; MBP, mean blood pressure; SpO2, peripheral blood oxygen saturation; AIDS: acquired immune deficiency dyndrome; COVID-19: coronavirus disease 2019; SOFA, sequential organ failure assessment; APS III, scute physiology score III; CCI, charlson comorbidity index; RBC, red blood cell; WBC, white blood cell; BUN, blood urea nitrogen; PT, prothrombin time; PTT, partial thromboplastin time.

Supplementary Table 5. Schoenfeld residual test for proportional hazards assumption in the fully adjusted cause- specific hazards model

Variable	chisq	df	P-value
SII (per 1000 units)	0.81	1	0.368
Sex	0.03	1	0.870
Age	6.34	1	0.012
Race	3.59	1	0.058
BMI	0.81	1	0.367
History of tobacco use	0.10	1	0.756
ICU type	9.63	1	0.002
Admission type	0.05	1	0.822
Days on IMV	21.8	1	<0.001
Days on RRT	1.24	1	0.266
Days on invasive line	6.62	1	0.010
Heart rate	0.001	1	0.979
Respiratory rate	0.008	1	0.930
Temperature	4.96	1	0.026
SpO_2	0.02	1	0.887
Blood glucose	0.009	1	0.923
AIDS	0.13	1	0.715
COVID-19	0.49	1	0.482
Diabetes	0.001	1	0.979
Cerebrovascular disease	11.7	1	<0.001
Metastatic solid tumor	1.32	1	0.250

Variable	chisq	df	P-value
Congestive heart failure	3.08	1	0.079
Chronic pulmonary disease	0.22	1	0.641
Rheumatic disease	1.06	1	0.303
Renal disease	1.02	1	0.313
Liver disease	0.11	1	0.738
SOFA score	1.26	1	0.261
SAPS II	1.57	1	0.210
WBC count	1.17	1	0.280
BUN	0.46	1	0.500
Creatinine	0.09	1	0.763
PT	6.38	1	0.012
PTT	0.63	1	0.427
GLOBAL	70.2	33	<0.001

Supplementary Table 6. Full Model and Sensitivity Analysis (Excluding WBC) for the Association between SII and ICU-AI

Variable	Full Model CSHR (95% CI) (P)*	Sensitivity CSHR (95% CI) (P)**	Full Model SHR (95% CI) (P)*	Sensitivity SHR (95% CI) (P)**
SII, $\times 10^9$/L (Pre 1000-unit)	1.06 (1.03~1.08) (<0.001)	1.06 (1.04–1.09) (P=0.000)	1.05 (1.03~1.07) (<0.001)	1.05 (1.03–1.08) (P=0.000)
SII quartile, $\times 10^9$/L				
Q1 (≤ 594)	1.0 (reference)	1.0 (reference)	1.0 (reference)	1.0 (reference)
Q2 (595-1124)	1.19 (0.91~1.56) (0.198)	1.24 (0.95–1.62) (P=0.115)	1.28 (0.99~1.66) (0.064)	1.34 (1.03–1.73) (P=0.030)
Q3 (1125-2201)	1.44 (1.11~1.86) (0.006)	1.48 (1.15–1.92) (P=0.003)	1.51 (1.17~1.93)(<0.001)	1.58 (1.23–2.02) (P=0.000)
Q4 (≥ 2202)	1.76 (1.37~2.26) (<0.001)	1.87 (1.46–2.38) (P=0.000)	1.96 (1.54~2.49) (<0.001)	2.08 (1.63–2.65) (P=0.000)

Variable	Full Model CSHR (95% CI) (P)*	Sensitivity CSHR (95% CI) (P)**	Full Model SHR (95% CI) (P)*	Sensitivity SHR (95% CI) (P)**
P for trend	<0.001	<0.001	<0.001	<0.001

*adjusted for age, race, sex, BMI, history of tobacco use, ICU type, admission type, treatment before infection (days on IMV, days on invasive line, days on RRT), vital signs (heart rate, respiratory rate, temperature, Spo2 and blood glucose), comorbidity (AIDS, COVID-19, diabetes, cerebrovascular disease, metastatic solid tumor, congestive heart failure, chronic pulmonary disease, rheumatic disease, renal disease and liver disease),SOFA score, SAPS II, laboratory parameters (WBC count, BUN, creatinine, PT and PTT)

**adjusted for variables in the Full Model minus WBC count

Supplementary Table 7. Sensitivity analysis comparing the association between SII and culture-positive ICU-acquired infection

Variable	Proportional cause-specific hazard model		Fine-Gray subdistribution hazard model	
	Crude CSHR (95%CI) (P-value)	Fully Adjusted CSHR * (95%CI) (P-value)	Crude SHR (95%CI)(P-value)	Fully Adjusted SHR * (95%CI) (P-value)
SII, $\times 10^9$/L (Pre 1000-unit)	1.07 (1.04–1.09) (P<0.001)	1.07 (1.04–1.09) (P<0.001)	1.05 (1.03~1.08) (<0.001)	1.05 (1.03–1.08) (P<0.001)
SII quartile, $\times 10^9$/L				
Q1 (≤ 594)	1.0 (reference)	1.0 (reference)	1.0 (reference)	1.0 (reference)
Q2 (595-1124)	1.18 (0.91–1.54) (P=0.209)	1.25 (0.96–1.63) (P=0.104)	1.26 (0.97–1.64) (P=0.081)	1.33 (1.02–1.73) (P=0.033)
Q3 (1125-2201)	1.27 (0.99–1.63) (P=0.056)	1.39 (1.07–1.79) (P=0.012)	1.68 (1.31–2.15) (P<0.001)	1.56 (1.22–2.01) (P<0.001)
Q4 (≥ 2202)	1.75 (1.38–2.21) (P=<0.001)	1.72 (1.34–2.20) (P=<0.001)	2.37 (1.88–3.00) (P<0.001)	2.06 (1.61–2.63) (P<0.001)
P for trend	<0.001	<0.001	<0.001	<0.001

*adjusted for age, race, sex, BMI, history of tobacco use, ICU type, admission type, treatment before infection (days on IMV, days on invasive line, days on RRT), vital signs (heart rate, respiratory rate, temperature, Spo2 and blood glucose), comorbidity (AIDS, COVID-19, diabetes, cerebrovascular disease, metastatic solid tumor, congestive heart failure, chronic pulmonary disease, rheumatic disease, renal disease and liver disease),SOFA score, SAPS II, laboratory parameters (WBC count, BUN, creatinine, PT and PTT)
